# Production of an EP/PDMS/SA/AlZnO Coated Superhydrophobic Surface through an Aerosol-Assisted Chemical Vapor Deposition Process

**DOI:** 10.1021/acs.langmuir.2c01060

**Published:** 2022-06-13

**Authors:** Seonghyeok Park, Jiatong Huo, Juhun Shin, Ki Joon Heo, Julie Jalila Kalmoni, Sanjayan Sathasivam, Gi Byoung Hwang, Claire J. Carmalt

**Affiliations:** † Materials Chemistry Research Centre, Department of Chemistry, University College London, 20 Gordon Street, London WC1H 0AJ, United Kingdom; ‡ School of Engineering, London South Bank University, 103 Borough Rd, London SE1 0AA, United Kingdom

## Abstract

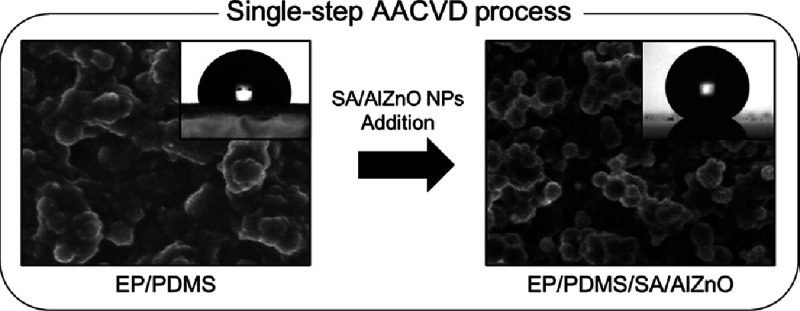

In this study, a superhydrophobic coating on glass has been prepared through a single-step aerosol-assisted chemical vapor deposition (AACVD) process. During the process, an aerosolized precursor containing polydimethylsiloxane, epoxy resin, and stearic acid functionalized Al-doped ZnO nanoparticles was deposited onto the glass at 350 °C. X-ray photoelectron spectroscopy, scanning electron microscopy, and atomic force microscopy showed that the precursor was successfully coated and formed a nano/microstructure (surface roughness: 378.0 ± 46.1 nm) on the glass surface. The coated surface had a water contact angle of 159.1 ± 1.2°, contact angle hysteresis of 2.2 ± 1.7°, and rolling off-angle of 1°, indicating that it was superhydrophobic. In the self-cleaning test of the coated surface at a tilted angle of 20°, it was shown that water droplets rolled and washed out dirt on the surface. The stability tests showed that the surface remained superhydrophobic after 120 h of exposure to ultraviolet (UV) irradiation and even after heat exposure at 350 °C. In addition, the surface was highly repellent to water solutions of pH 1–13. The results showed that the addition of the functionalized nanoparticles into the precursor allowed for the control of surface roughness and provided a simplified single-step fabrication process of the superhydrophobic surface. This provides valuable information for developing the manufacturing process for superhydrophobic surfaces.

## Introduction

A superhydrophobic surface is defined by a phenomenon where a water droplet on a surface gives a water contact angle of >150°, contact angle hysteresis of <10°, and rolling off-angle of <10°.^1−3^ This comes from two key properties of a surface, namely, low surface energy and nano- and micro-scale surface roughness.^4^ Both conditions must be satisfied for the surface to be superhydrophobic. In nature, the lotus leaf is one of the most well-known examples of a superhydrophobic surface.^5−8^ The hydrophobic nano/microstructure on the surface allows the leaf to keep dry and clean by droplets collecting dust as they roll off on the surface.^5−8^ It is also found on the wings and legs of insects.^9−12^ Butterfly and cicada use the water repellent feature to prevent their wings from accumulating dust and wetting,^9,13^ and the hydrophobic legs of water striders make these insects capable of floating on the water surface.^14,15^ It was reported that superhydrophobic surfaces could have self-cleaning, anti-biofouling, anti-corrosion, and anti-icing/fogging features.^16−18^ These novel properties led many researchers to develop artificial superhydrophobic surfaces.^16−23^

Various techniques, including spin-coating, surface etching, and dip-coating methods, have been investigated to produce superhydrophobic surfaces.^24−27^ Dip-coating is a type of liquid-phase deposition.^28−32^ It can be easily applied to various substrates, including glass, cotton, steel mesh, and plastic surfaces.^28−32^ Qing *et al.* reported a titanium dioxide (TiO_2_) nanoparticle (NP)/polydimethylsiloxane (PDMS) coating onto a copper surface using the dipping process resulting in a superhydrophobic surface with a water contact angle of 162.3° and rolling off-angle of 4.3°.^31^ Han *et al.* showed that dipping a polyester membrane into a mixture of silicon dioxide (SiO_2_) NPs and PDMS produced a highly hydrophobic surface with a water contact angle of 162°.^29^ Spin-coating, another liquid-phase deposition method, is a process to uniformly deposit a solution across a surface by centrifugal force, and its coating thickness can be controlled by the angular speed of spinning.^33−35^ Meena *et al.* showed that a three-times coating of a solution containing polymethylmethacrylate, hexadecyltrimethoxysilane, and SiO_2_ NPs onto a glass surface produced a superhydrophobic surface with a water contact angle of 165° and a rolling off-angle of 7°.^34^ Similarly, Long *et al.* showed that a PDMS coating onto a rough aluminum surface at 3000 rpm changed the surface from a hydrophilic to a highly hydrophobic surface.^35^

The aerosol-assisted chemical vapor deposition (AACVD) process is an easy and scalable technique. The process does not need volatile, but soluble precursors in any solvent from which aerosols are generated. This provides more excellent capability and flexibility to produce films and coatings than traditional chemical vapor deposition.^36^ The technique has been widely used to study superhydrophobic surfaces.^27,37−39^ The aerosolized precursor is delivered and deposited to a substrate in the AACVD chamber during the process.^36^ Depending on the type of precursor, single or multiple deposition processes have been used to produce superhydrophobic surfaces.^27,37−39^ For example, Tombesi *et al*. introduced a layer-by-layer deposition using 3-methacryloxypropyltrimethoxysilane and tetraethyl orthosilicate (TEOS) while Zhuang *et al.* described a single-step deposition using PDMS and TEOS.^38,40^ This study introduces a single-step AACVD process using a precursor mixture containing PDMS, epoxy resin, and stearic acid functionalized Al-doped ZnO nanoparticles to produce a superhydrophobic surface. The addition of the functionalized nanoparticles into the precursor solution containing PDMS and epoxy resin allowed for the control of surface roughness and provided a simplified single-step fabrication process of the superhydrophobic surface. The coating produced by the process at 350 °C showed superhydrophobicity with high water repellency and self-cleaning features, as well as good stability against UV irradiation, heat ranging from 20 to 350 °C, and various levels of water pH.

## Materials and Methods

The Sylgard-184 Elastomer kit with a curing agent (PDMS) was purchased from Dow Corning. Bisphenol A diglycidyl ether and triethylenetetramine, 6% Al-doped ZnO nanoparticles (50 nm in size), stearic acid, and methanol were purchased from Sigma Aldrich. Ethyl acetate was purchased from Fisher Scientific. All reagents were used without further purification steps.

### Synthesis of Stearic Acid Functionalized Al-Doped ZnO Nanoparticles

A total of 0.35 g of stearic acid was dissolved in 50 mL of ethanol and then stirred at 70 °C for 2 h. A total of 1 g of Al-doped ZnO nanoparticles was mixed with the stearic acid solution and then sonicated for 5 min. The solution was centrifuged at 4500 rpm for 5 min and washed with ethanol three times. The collected particles were dried at 200 °C for 24 h.

### Precursor Preparation

A total of 400 mg of PDMS and 40 mg of curing agent were dissolved in 40 mL of ethyl acetate and stirred for 10 min to produce the PDMS solution. A total of 600 mg of bisphenol A diglycidyl ether and 60 mg of triethylenetetramine were dissolved in 20 mL of methanol and stirred for 10 min to produce the epoxy resin (EP) solution. The PDMS and EP solutions were mixed, and 10 wt % of SA functionalized AlZnO NPs were dispersed in the mixture and then sonicated for 10 min to produce the overall precursor mixture. This precursor mixture was used to produce the superhydrophobic surfaces.

### Precursor Deposition through the AACVD Process

Before the AACVD process, the glass substrate (145 mm × 35 mm × 5 mm) was washed with acetone and isopropanol, dried in an oven, and then cleaned using an air plasma cleaner for 5 min to remove contaminants on the surface. The 60 mL of the precursor was loaded into the AACVD bubbler and was aerosolized using an ultrasonic humidifier. N_2_ carrier gas was supplied at a flow rate of 0.8 L/min at 350 °C. Precursor deposition on the substrate was carried out for 10, 20, and 40 min. After that, the reactor and nitrogen flow were turned off, and the coated samples were cooled down to room temperature.

### ATR-FTIR Spectroscopy

An attenuated total reflection (ATR)-Fourier transform infrared (FTIR) spectrometer was used to characterize the stearic acid (SA), Al-doped ZnO (AlZnO) nanoparticles (NP), and stearic acid caped Al-doped ZnO nanoparticles (SA/AlZnO NPs).

### Water Contact Angle Measurement

Water contact angle, rolling off-angle, and contact angle hysteresis against the samples were measured using an FTA-1000B drop shape instrument (First Ten Angstroms Inc., VA, USA). The measurements were conducted at three different places for each sample. To measure the water contact angle, 10 μL of DI water was put onto the sample surface, it was photographed side on, and the images were analyzed using ImageJ. The rolling off-angle of the water droplet was measured at a tilted angle of the sample from 1 to 90°. The contact angle hysteresis was measured by the difference between the advancing and receding angles of water droplets.^32^

### AFM and UV–vis Transmittance Analysis

The roughness of the sample surfaces (10 μm × 10 μm) was measured using atomic force microscopy (AFM, NaniteAFM, Liestal, Switzerland). Electrostatic force microscopy (EFM) mode and dynamic force mode were employed for the measurement, and the resonant frequency of the cantilever ranged from 150 to 200 kHz. UV–vis transmittance spectra of the samples were measured using a Lambda 950 spectrometer (PerkinElmer Inc., Winter St., CT, USA), which has a detection range of wavelength 185–3100 nm. The transmittance of the samples was measured in a wavelength range of 250–800 nm.

### X-ray Photoelectron Spectroscopy and SEM Analysis

X-ray photoelectron spectroscopy (XPS) data was taken using a ThermoScientific X-ray photoelectron spectrometer with a monochromatic Al Kα X-ray source (1486.96 eV), and depth profiling using an argon gun was performed for 0 (sample surface) and 200 (inside the sample) s. To determine the surface topography of the samples, scanning electron microscopy (SEM, JEOL Inc., Peabody, MA, USA) was employed. To inhibit surface charging, the sample was coated with gold crystals for 60 s through a sputter coating process, and then the topography was observed by SEM at an accelerating voltage of 5 kV.

### UV Stability Test of the Superhydrophobic Surface

Superhydrophobic samples were exposed to ultraviolet (UV) light for 120 h. The superhydrophobic sample was placed in a wooden box with a UV lamp (emission wavelength: 365 nm, intensity: 3.7 mW/cm^2^). The distance between the sample and the lamp was about 20 cm. The water contact angle, contact angle hysteresis, and rolling off-angle were measured at intervals of 24 h.

### Heat Stability Test of the Superhydrophobic Surface

The superhydrophobic sample was placed in a furnace and exposed to heat energy ranging from 20 to 350 °C. At each condition, the sample was exposed to heat for 30 min. After that, the sample was allowed to cool down to room temperature, and the water contact angle, contact angle hysteresis, and rolling off-angle were measured.

### pH Stability Test of the Superhydrophobic Surface

For pH control, deionized (DI) water, sodium hydroxide (NaOH), hydrochloric acid (HCl), and a pH meter were used. NaOH and HCl were added to DI water for >pH 7 and <pH 7, respectively. Water contact angle, contact angle hysteresis, and rolling off-angle of the samples were measured using the water solutions of pH 1–13.

## Results and Discussion

**Figure 1:**
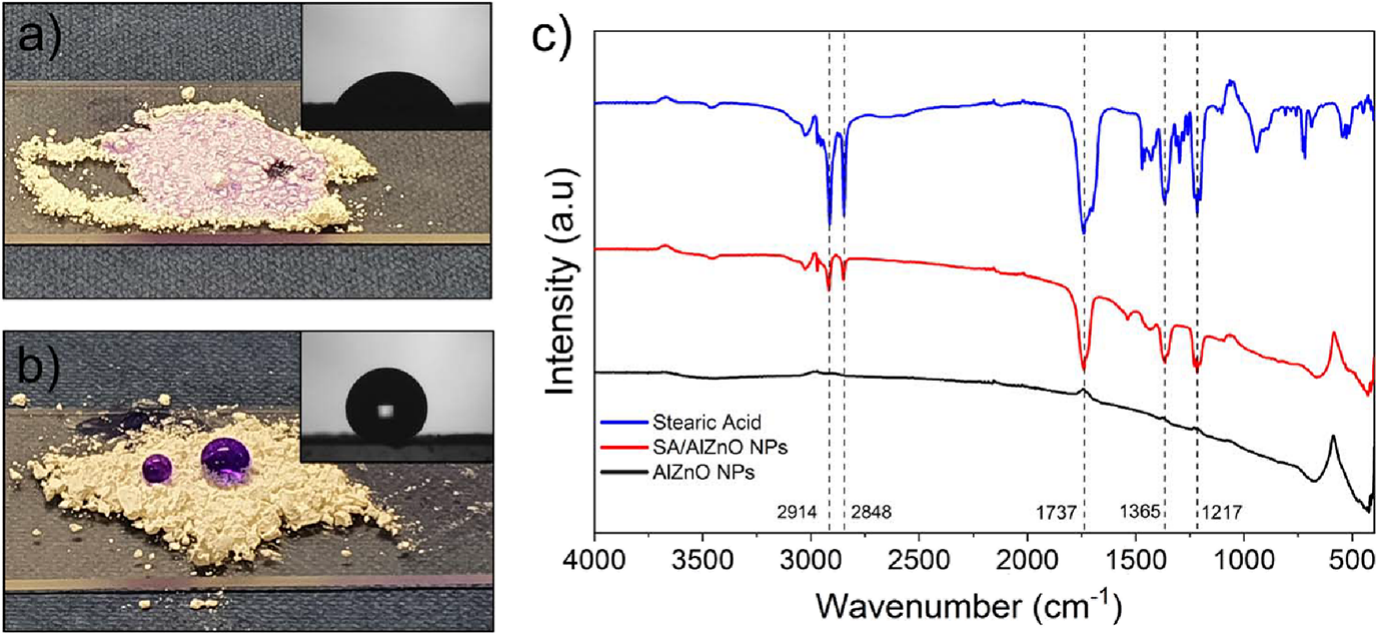
Water repellent test of (a) Al-doped ZnO nanoparticles (AlZnO NPs only) and (b) stearic acid (SA) functionalized AlZnO NPs (SA/AlZnO NPs); (c) infrared spectra of SA/AlZnO NPs, SA, and AlZnO NPs in the wavenumber range of 400 to 4000 cm^–1^.

To produce highly hydrophobic nanoparticles, 6% Al-doped ZnO nanoparticles were mixed in an ethanol solution containing stearic acid (SA) and then collected by centrifugation and dried at 200 °C. As shown in Figure 1a,b, stearic acid treatment transformed the AlZnO NPs from hydrophilic to highly hydrophobic, and the purple colored water droplets formed a sphere on the SA/AlZnO NPs. Figure 1c shows the infrared spectra of SA/AlZnO NPs, SA, and AlZnO NPs in the range of 400 to 4000 cm^–1^. Peaks at 1217, 1365, 1737, 2848, and 2914 cm^–1^ were observed for the SA/AlZnO NPs, indicating the presence of SA molecules. The peaks at 2848 and 2914 cm^–1^ can be attributed to the alkane C–H symmetric stretching of stearic acid, and the peak at 1737 cm^–1^ corresponds to the carboxylic acid C=O stretch.^41,42^ As expected, peaks corresponding to SA were not observed on the uncoated AlZnO NPs. To produce a precursor for the AACVD process, the hydrophobic SA/AlZnO NPs were added to the PDMS/EP solution. When AlZnO NPs, which are hydrophilic, were added to the mixture, the nanoparticles agglomerated in the solution, resulting in an unusable mixture. However, SA bonding to the AlZnO NPs addressed the dispersion issue. The highly hydrophobic nanoparticles were uniformly dispersed in the mixture.

**Figure 2:**
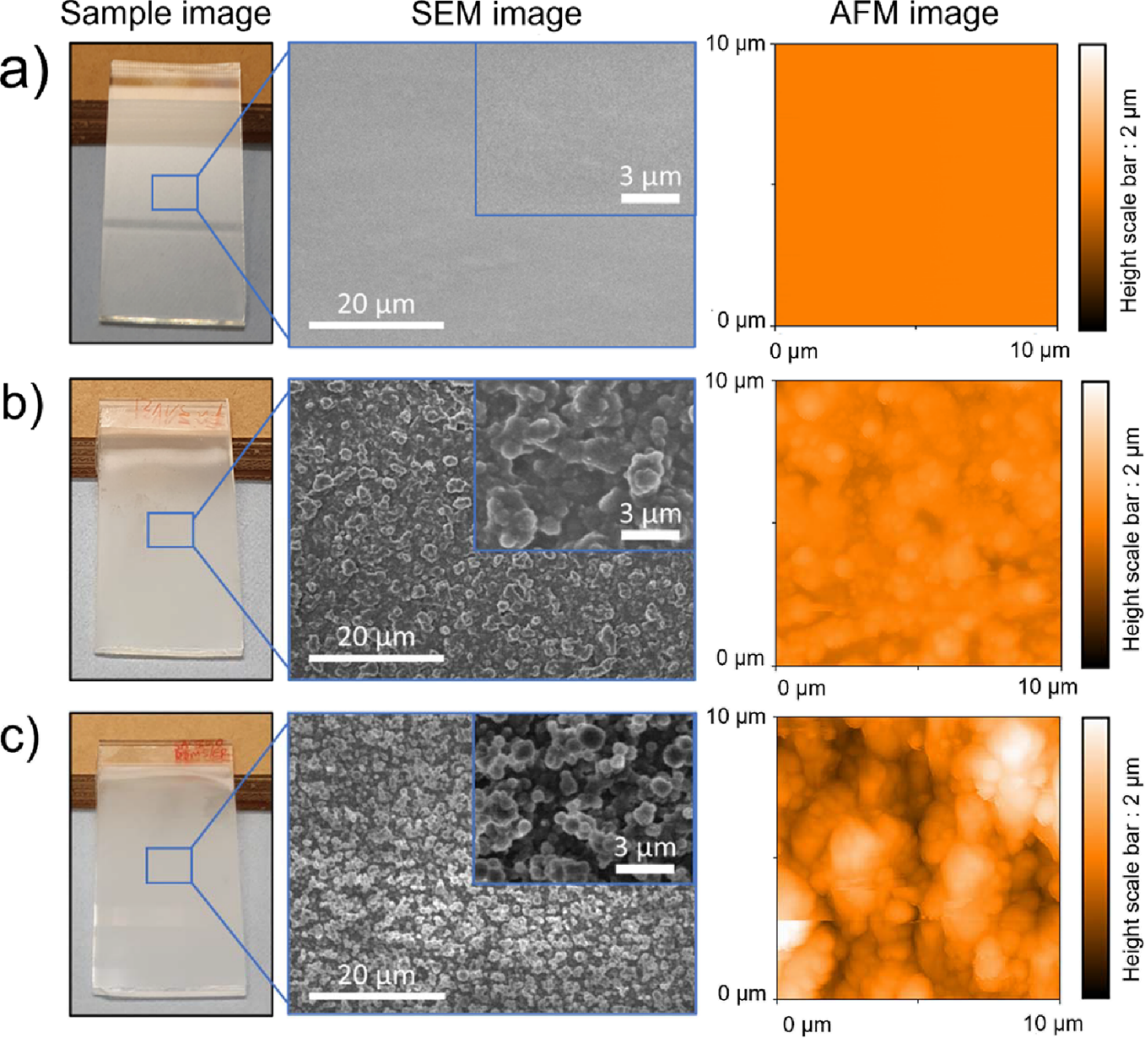
Photo images, SEM images, and AFM images of the (a) glass substrate, (b) EP/PDMS, and (c) EP/PDMS/SA/AlZnO samples.

The thermal degradation of PDMS occurs at >350 °C,^43^ and in the AACVD process, a high temperature is necessary to provide the required energy for a chemical reaction between the precursor and substrate, resulting in film growth.^36^ Considering these factors, the aerosolized precursor was deposited on a glass substrate at 350 °C, to avoid degradation of the PDMS. As shown in Figure S1, the surface roughness of the samples increased with increasing the deposition time, resulting in a significant increase in water contact angle. It was observed that the 40 min deposition was optimal for producing a superhydrophobic surface. Figure 2 shows surface images of the intact glass, EP/PDMS, and EP/PDMS/SA/AlZnO samples. After 40 min, the coating of EP/PDMS and EP/PDMS/SA/AlZnO resulted in the transparent glass being altered to a semi-transparent color. The appearances of the EP/PDMS and PDMS/SA/AlZnO coated substrates were similar. However, SEM, AFM, and light transmittance analyses showed a clear difference between the two coatings (Figure S2). The samples had different surface topographies. It was observed that the polymer particles formed microstructures on the EP/PDMS coating. This can be explained by the fact that during the deposition process, the fine precursor droplets were solidified and aggregated on the heated glass substrate at the same time, resulting in microstructure formation. Compared to the EP/PDMS film, more particles on the PDMS/SA/AlZnO coating were aggregated and stacked on top of each other, creating “mountain-like” nano/microstructures. The surface roughness (root mean square of area roughness: *S_q_*) of the plain glass substrate was 1.4 ± 0.4 nm, which increased to 109.4 ± 27.2 nm for the EP/PDMS film. A significant increase in surface roughness was observed for the EP/PDMS/SA/AlZnO films with an *S_q_* of 378.0 ± 46.1 nm, which provides an indication of how the presence of the NPs enhanced the surface roughness. The light transmittance test showed that the plain glass substrate had a transmittance of >91% in the visible range, while the EP/PDMS and EP/PDMS/SA/AlZnO coatings showed transmittances of 85–90 and 80–82%, respectively (Figure S2).

**Figure 3:**
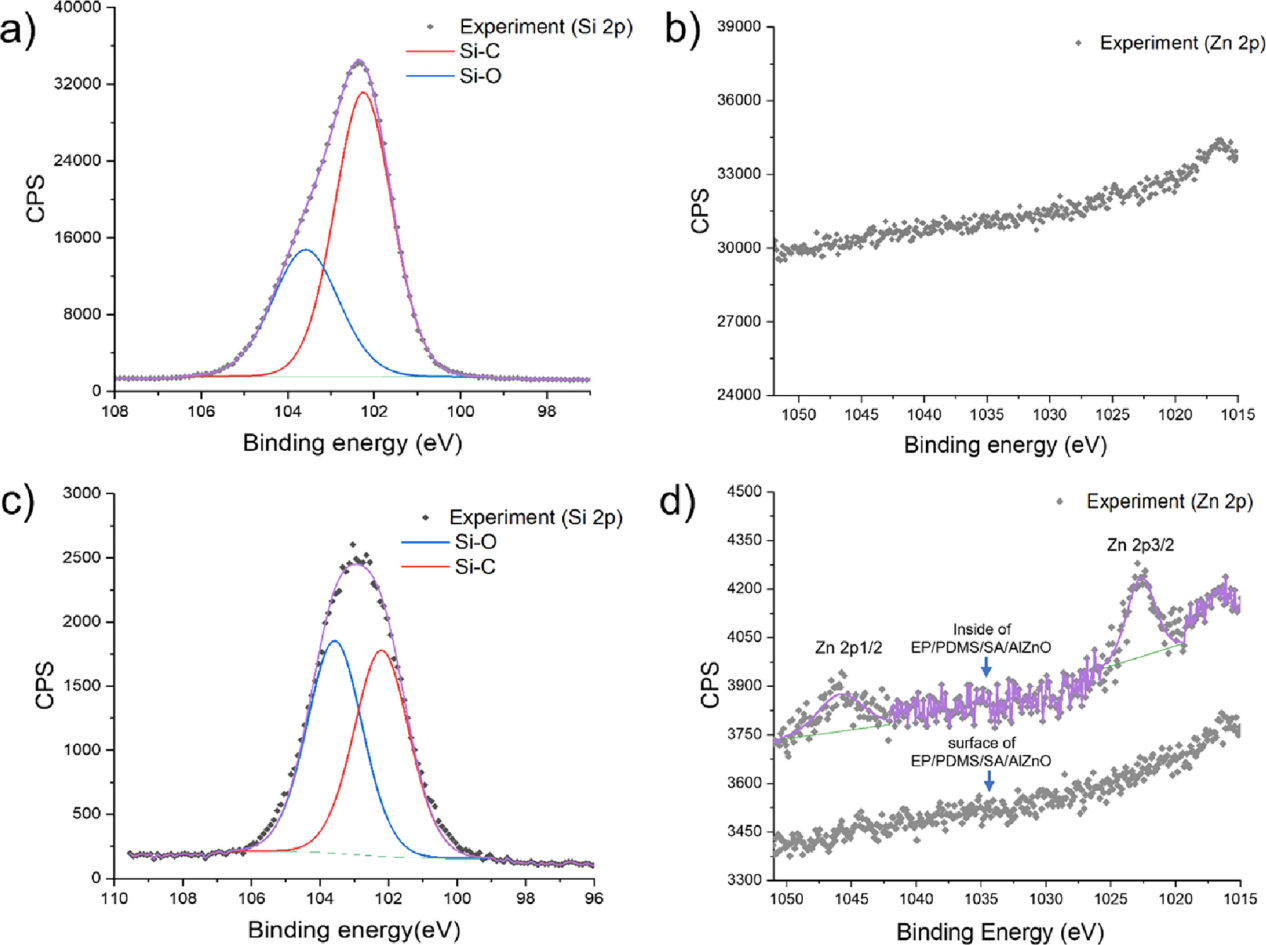
High resolution XPS spectra of Si 2p and Zn 2p of (a, b) EP/PDMS and (c, d) EP/PDMS/SA/AlZnO samples.

XPS was performed to determine the surface chemistry of the EP/PDMS and EP/PDMS/SA/AlZnO coatings, and an adventitious carbon peak at 284.6 eV was used as a charge reference in the XPS analysis. As shown in Figure 3a,c, a broad Si 2p spectrum was observed for the EP/PDMS and EP/PDMS/SA/AlZnO samples, and the spectrum can be deconvoluted into two peaks, indicating Si–C (at a binding energy of 102.2 eV) and Si–O (at a binding energy of 103.5 eV) bonds within the PDMS molecule.^44^ As expected, no peaks due to Zn were observed for the EP/PDMS sample. For the EP/PDMS/SA/AlZnO sample, peaks corresponding to Zn were also not observed on the surface, but the Zn 2p peak was detected in the bulk of the film (Figure 3b,d). The Zn 2p shows a double peak at 1021.8 (Zn 2p3/2) and 1045.1 eV (Zn 2p1/2), indicating the existence of SA/AlZnO NPs.^45^

**Figure 4:**
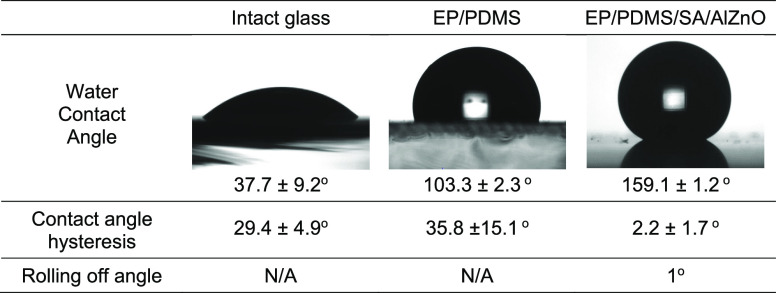
Water contact angle, contact angle hysteresis, rolling off-angle of intact glass, EP/PDMS, and EP/PDMS/SA/AlZnO samples.

Figure 4 shows the water contact angle, contact angle hysteresis, and rolling off-angle of the glass substrate and the EP/PDMS and the EP/PDMS/SA/AlZnO coatings. The glass substrate gave a water contact angle of 37.7 ± 9.2° and contact angle hysteresis of 29.4 ± 4.9°, whereas the contact angle and hysteresis of the EP/PDMS coating were 103.3 ± 2.3° and 35.8 ± 15.1°, respectively. In the rolling off test against the uncoated glass substrate and the EP/PDMS samples, rolling of the water droplet (10 μL) was not observed, and the droplet stayed on the sample surfaces even at a tilt angle of 90°. In contrast with these samples, the EP/PDMS/SA/AlZnO sample surface was highly water repellent. The contact angle and hysteresis of the surface were 159.1 ± 1.2° and 2.2 ± 1.7°, respectively, and the water droplet rolled off the surface at a tilted angle of 1°, indicating that the EP/PDMS/SA/AlZnO film showed superhydrophobic properties. The Wenzel and Cassie–Baxter laws are the main models to explain the wetting of surface roughness.^46^ The Wenzel model is related to a homogeneous regime where liquid penetrates the grooves of rough surfaces.^46,47^ In contrast, the Cassie–Baxter model is associated with a heterogeneous regime where air bubbles are entrapped in the grooves.^46,48^ In this study, the EP/PDMS/SA/AlZnO coating is a heterogeneous surface. Figure S3 shows that a change in water contact angle of the coating by the surface roughness complied with the Cassie–Baxter model, indicating that the EP/PDMS/SA/AlZnO coating is superhydrophobic in the Cassie–Baxter state.

**Figure 5:**
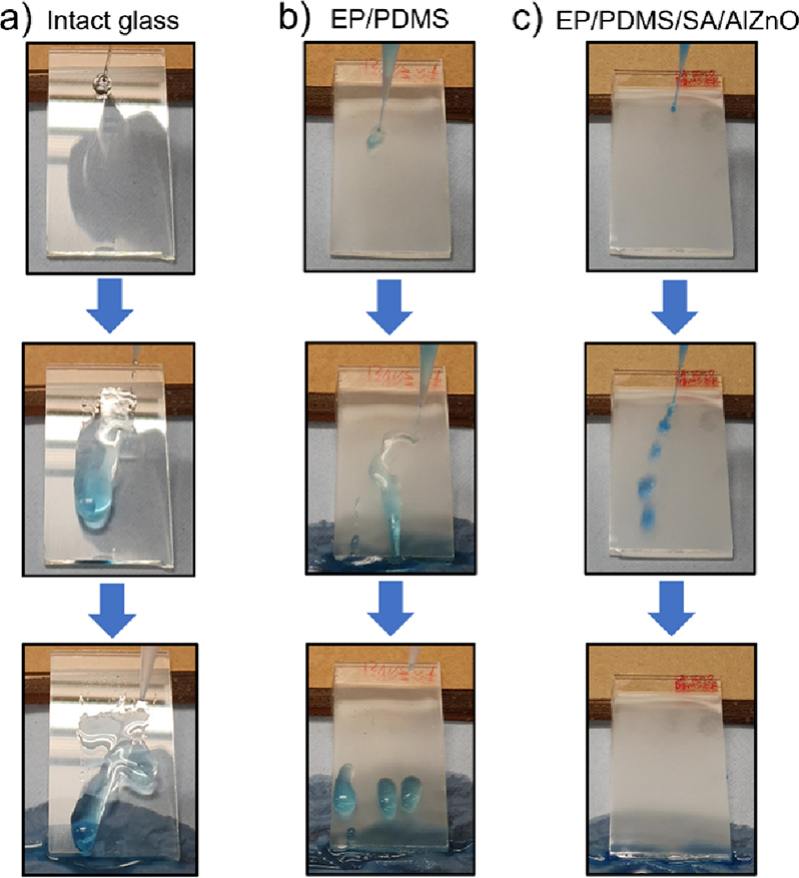
Water repellent test of (a) intact glass, (b) EP/PDMS, and (c) EP/PDMS/SA/AlZnO samples at a tilted angle of 20°.

**Figure 6:**
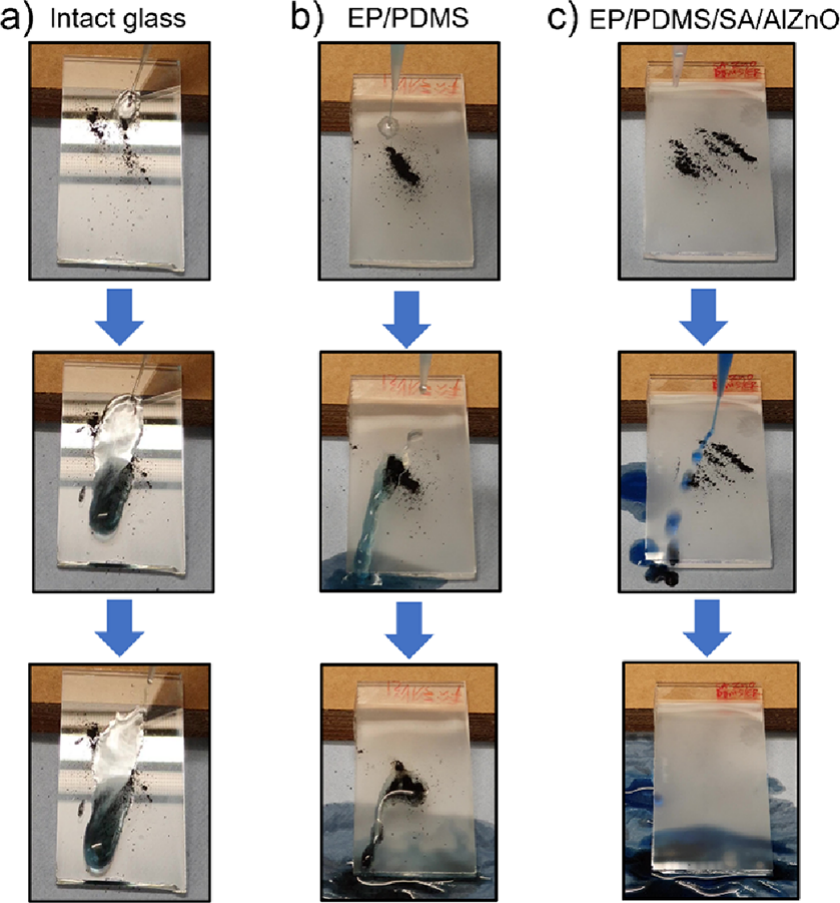
Self-cleaning tests of (a) intact glass, (b) EP/PDMS, and (c) EP/PDMS/SA/AlZnO surfaces at a tilted angle of 20°.

The water repellent and self-cleaning tests of plain glass, EP/PDMS, and EP/PDMS/SA/AlZnO samples were performed at a titled angle of 20°. In the test, multiple water droplets containing methylene blue dye were continuously dropped onto the surfaces at a distance of 1 cm. As shown in Figure 5, the dropped water droplets were trapped on the surface of the plain glass and the EP/PDMS coated substrate, although excessive water slid off both samples. In contrast, the water droplets readily rolled off on the EP/PDMS/SA/AlZnO sample without wetting the surface. For the self-cleaning test, graphene oxide powder was used as dirt. As shown in Figure 6, the self-cleaning experiment showed that after water dropping, the dirt within the water remained on the glass and the EP/PDMS surface, whereas the dirt was carried away when the droplets rolled off the surface of the EP/PDMS/SA/AlZnO coating. This self-cleaning feature of the EP/PDMS/SA/AlZnO sample can be explained by the high roughness with low surface energy decreasing the contact between the water droplet and sample surface, resulting in a significant reduction of adhesion force between the surface and water. Because the adhesion of water droplets to dirt was much higher than that of the surface, the water droplets washed away the dirt.

**Figure 7:**
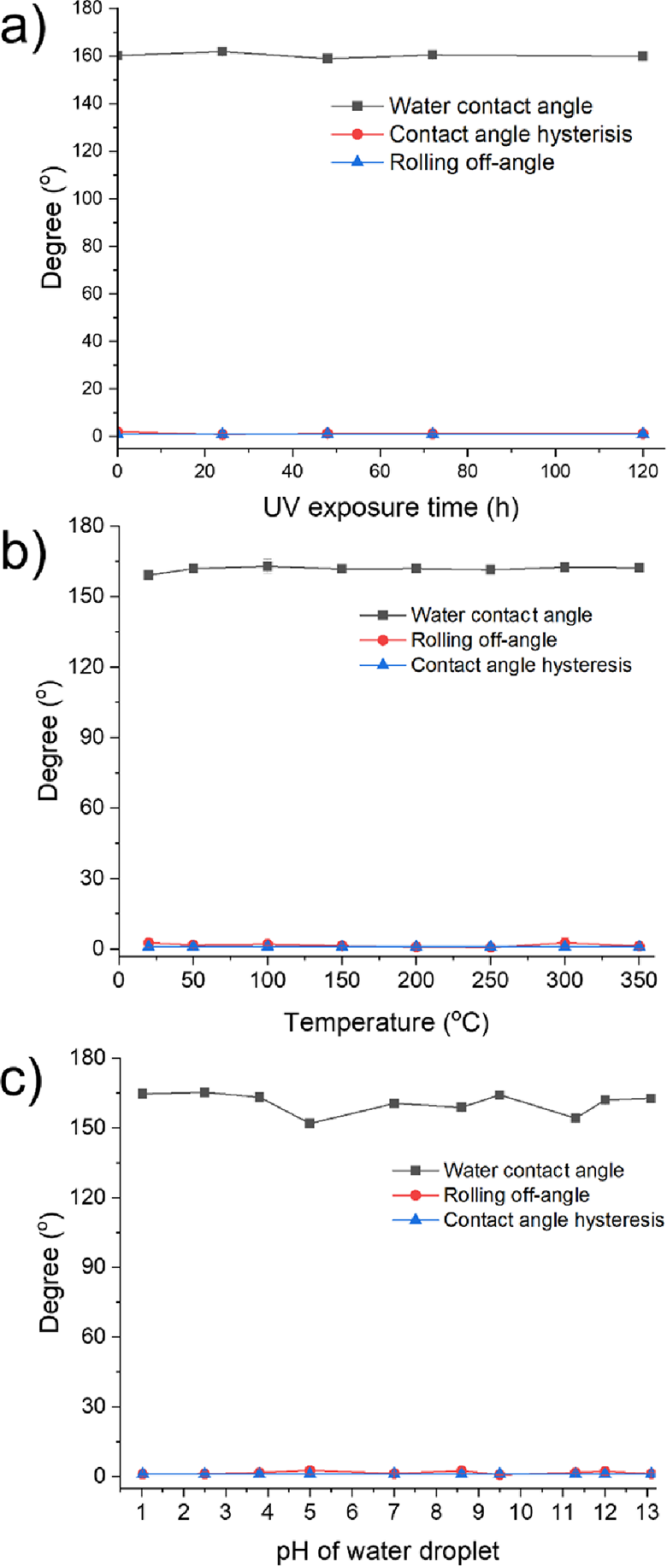
Stability of water contact, contact angle hysteresis, and rolling off-angle of EP/PDMS/SA/AlZnO surfaces in terms of (a) UV(365 nm) irradiation for 120 h, (b) heat (20 to 350 °C), and (c) water solutions of pH 1–13.

Figure 7 shows the stability of the EP/PDMS/SA/AlZnO sample against UV irradiation, heat, and various levels of water pH. In the UV test, the sample was exposed to a UV light source with an emission wavelength of 365 nm. AlZnO nanoparticles within the superhydrophobic coating are UV-activated photocatalysts inducing reactive oxygen species (ROS). It was reported that ROS could degrade organic compounds.^49,50^ Thus, a UV stability test was performed to determine if the UV irradiation negatively affected the sample’s superhydrophobicity. As shown in Figure 7a, a change in water contact angle, contact angle hysteresis, and rolling off-angle was not observed after UV exposure over 120 h, indicating that UV irradiation did not affect the superhydrophobic properties. In Figure 7b, the thermal stability test showed that despite heat exposure up to 350 °C, the coating remained superhydrophobic, indicating that the EP/PDMS/SA/AlZnO coating was stable at 350 °C. In the pH test, the sample repellency to water solutions of pH 1–13 was measured. The water contact angle of the sample was slightly affected by the pH levels, but it still kept a water contact angle of >150° with a contact angle hysteresis and rolling off-angle of <5° (Figure 7c).

EP and PDMS are hydrophobic adhesives, and PDMS is also commonly used as a surface energy lowering agent for producing a superhydrophobic surface in the AACVD processes.^40,51^ This study employed a single deposition using AACVD, and a precursor solution containing PDMS and epoxy resin was used. The precursor deposition on the glass substrate for 40 min significantly increased the water contact angle. However, it was not superhydrophobic. This is because the surface roughness of the EP/PDMS sample was not high enough to produce superhydrophobicity. The addition of the hydrophobic SA/AlZnO NPs into the coating significantly increased the surface roughness of the EP/PDMS surface. As a result, the water contact angle reached >150°, and rolling off- and hysteresis angles were <5°, indicating that the surface was superhydrophobic.

In previous studies, EP/PDMS depositions using the AACVD process were reported to produce a superhydrophobic surface. Guo *et al.* employed multi-layer depositions of EP/PDMS at 290 to 350 °C.^51^ It was shown that the water contact angle increased with increasing number of deposition cycles, and after three cycles, the surface became superhydrophobic with a contact angle of 163.9° and rolling off-angle of 1.7°.^51^ Zhuang *et al.* used a two-step AACVD process between 290 and 350 °C, producing a superhydrophobic surface with a contact angle of 160° and a rolling off-angle of <1°.^40^ It was shown that a single deposition of EP/PDMS on the glass substrate through AACVD did not obtain superhydrophobicity because the nano/microstructures on the surface did not sufficiently form and produce a high surface roughness.^51^ Zhuang *et al.* and Guo *et al.* used multiple depositions to address this issue, requiring a prolonged process time.^40,51^ In this study, the hydrophobic SA/AlZnO NPs were used to control the surface roughness of EP/PDMS, and a single deposition process using AACVD could produce a superhydrophobic surface.

## Conclusions

In this study, a composite of polydimethylsiloxane, epoxy resin, and stearic acid functionalized Al-doped ZnO nanoparticles was coated to the glass substrate through an AACVD process at 350 °C. The water repellent test showed that the composite deposited sample had a water contact angle of 159.1°, contact angle hysteresis of 2.2°, and rolling off-angle of <1°, indicating that it was superhydrophobic. The surface maintained superhydrophobicity after 120 h exposure to UV irradiation and even after heat exposure at 350 °C. In addition, the surface was superhydrophobic against water solutions of pH 1–13. Previous studies showed that in the AACVD process, multiple depositions were required when the precursor mixture containing polydimethylsiloxane and epoxy resin was used to produce a superhydrophobic surface.^35,38^ This study showed that adding the hydrophobic nanoparticles into the precursor provided a single-step AACVD process to produce a superhydrophobic surface, resulting in a simplified process. This study provides valuable information for developing the manufacturing process for superhydrophobic surfaces.

## Supporting Information Available

Surface topography, roughness, and water contact angle after 10, 20, and 40 min of deposition of precursor; UV–vis transmittance spectra of intact glass, EP/PDMS, and EP/PDMS/SA/AlZnO samples; and application of the Cassie–Baxter model to the water contact angle of EP/PDMS/SA/AlZnO surfaces (PDF)

## Abbreviations

AACVD: aerosol-assisted chemical vapor deposition
AFM: atomic force microscopy
AlZnO: Al-doped ZnO
ATR: attenuated total reflection
EFM: electrostatic force microscopy
EP: epoxy resin
NPs: nanoparticles
PDMS: polydimethylsiloxane
SA: stearic acid
SiO_2_: silicon dioxide
TEOS: tetraethyl orthosilicate
TiO_2_: titanium dioxide
XPS: X-ray photoelectron spectroscopy
UV: ultraviolet
