# Supplementary material for: Production of an EP/PDMS/SA/AlZnO Coated Superhydrophobic Surface through an Aerosol-Assisted Chemical Vapor Deposition Process
Source: Langmuir. 2022 Jun 13;38(25):7825–32. doi: 10.1021/acs.langmuir.2c01060 (PMC9245182; doi:10.1021/acs.langmuir.2c01060)
Supplement: Supplementary file 1 — la2c01060_si_001.pdf [file la2c01060_si_001.pdf]

<u>Supporting information</u>

Production of EP/PDMS/SA/AlZnO Coated Superhydrophobic Surface through an Aerosol-Assisted Chemical Vapour Deposition Process

Seonghyeok Park[a], Jiatong Huo[a], Juhun Shin[a], Ki Joon Heo[a], Julie Jalila Kalmoni[a], Sanjayan Sathasivam[a, b], Gi Byoung Hwang[a*] and Claire J. Carmalt[a*]

[a]*Materials Chemistry Research Centre, Department of Chemistry, University College London, 20 Gordon Street, London, WC1H 0AJ, United Kingdom*

[b]*School of Engineering, London South Bank University, 103 Borough Rd, London, SE1 0AA, United Kingdom*

E-mail: gi-byoung.hwang.14@ucl.ac.uk, c.j.carmalt@ucl.ac.uk

Table of Contents

Section I. Surface topography, roughness, and water contact angle after 10, 20, 40 min deposition of precursor ---S2

Section II. UV-vis transmittance spectra of intact glass, EP/PDMS and EP/PDMS/SA/AlZnO samples --S3

Section III. Application of the Cassie-Baxter model to the water contact angle of EP/PDMS/SA/AlZnO surfaces ---S4

Section I. Surface topography, roughness, and water contact angle after 10, 20, 40 min deposition of precursor

Figures S1 (a) and (b) showed surface topography and roughness after 10, 20, and 40 min depositions of the EP/PDMS/SA/AlZnO precursor at 350 ^{o}C. It was observed that microstructure and surface roughness on glass surfaces increased with increasing deposition time. The surface roughness and microstructure were the most prominent at the 40 min deposition. Figure S1 (c) shows a correlation between surface roughness and water contact angle. It was statistically confirmed that an increment in the surface roughness of the hydrophobic coating resulted in the increment in water contact angle (correlation coefficient: r = 0.91). At a surface roughness (S_q) of 378.0 ± 46.1 nm, the coating became superhydrophobic.

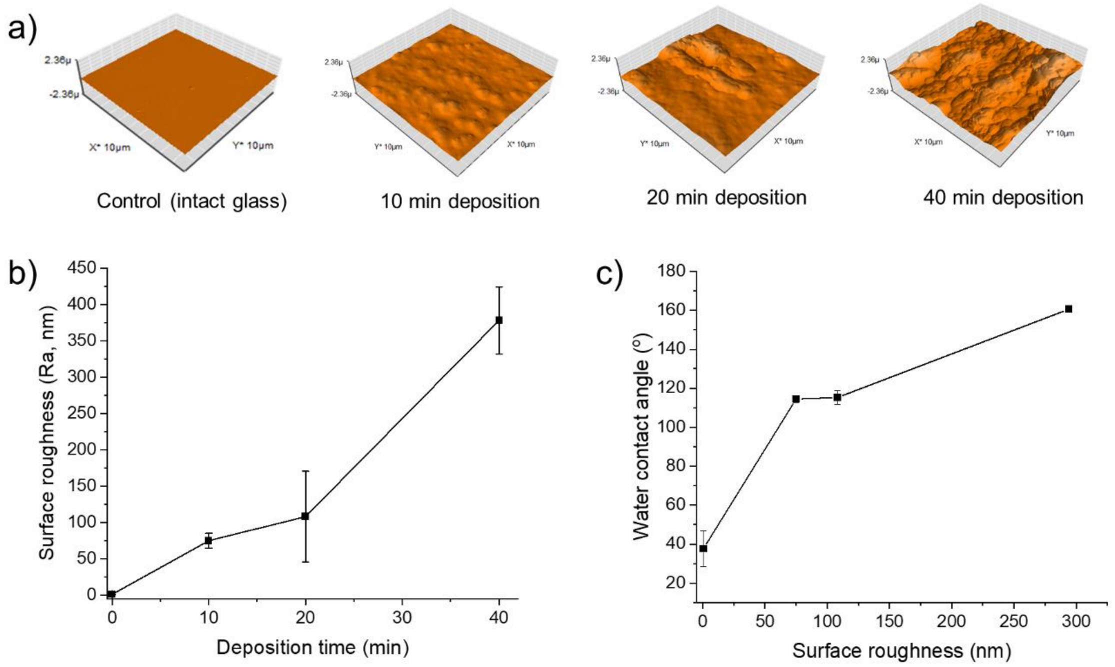

Figure S1. (a) Surface topography, (b) roughness of control (intact glass) and samples after 10, 20, 40 min deposition of precursor (EP/PDMS/SA/AlZnO), and (c) change in water contact angle with increasing surface roughness. The correlation between water contact angle and surface roughness was analysed by MS Excel software.

Section II. UV-vis transmittance spectra of intact glass, EP/PDMS and EP/PDMS/SA/AlZnO samples

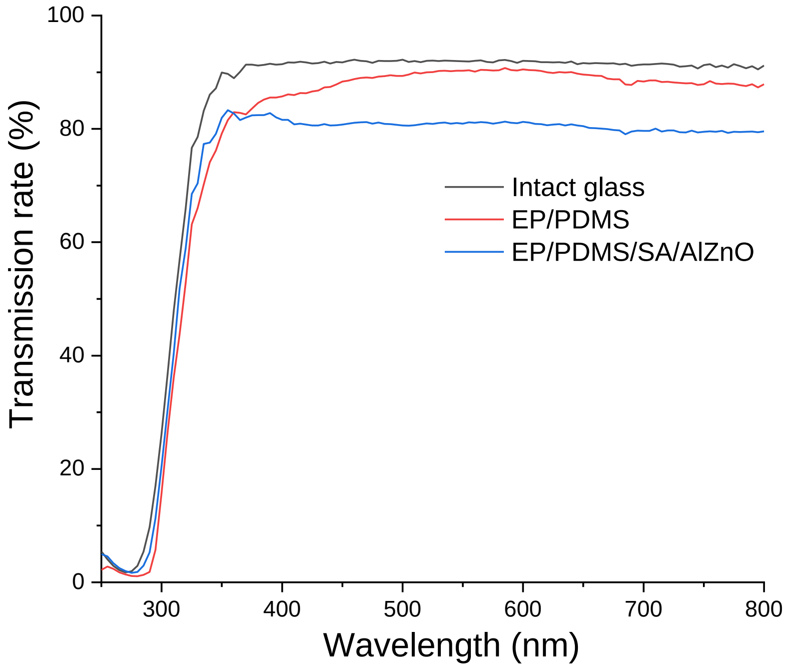

Figure S2. UV-vis transmittance spectra of intact glass, EP/PDMS and EP/PDMS/SA/AlZnO samples

Section III. Application of the Cassie-Baxter model to the water contact angle of EP/PDMS/SA/AlZnO surfaces

The correlation of the water contact angles between smooth and rough EP/PDMS/SA/AlZnO surfaces was determined. For rough surfaces, 10, 20, and 40 min deposited EP/PDMS/SA/AlZnO samples were used (Figure S1). To produce a smooth surface, a dip coating process was employed. A glass slide was dipped into a precursor containing polydimethylsiloxane, epoxy resin and stearic acid functionalised Al-doped ZnO nanoparticles for 3 sec, and it was withdrawn slowly, resulting in a formation of thin film on the glass surface. The coated surface was dried at 100 ^{o}C for 2 h. Angle measurement and AFM analysis showed that the water contact angle and roughness of the smooth EP/PDMS/SA/AlZnO surface were 108± 5.6^{o} and 12.7 ± 2.6 nm, respectively. Figure S3 shows the relationship of the water contact angle between the smooth and rough surfaces. The solid lines were drawn by changing the function of the solid/liquid contact area in the Cassie-Baxter equation below.

$$\mathrm{Cos}\theta_r = f\,(1+\mathrm{Cos}\theta_s) - 1$$

Where is f is the fraction of solid/liquid contact interface θ_r is the water contact angle on the rough surface and θ_s is the contact angle of a smooth surface. As shown in the figure, the angle change of EP/PDMS/SA/AlZnO surface by the increment of surface roughness well complies with the Cassie-Baxter model.

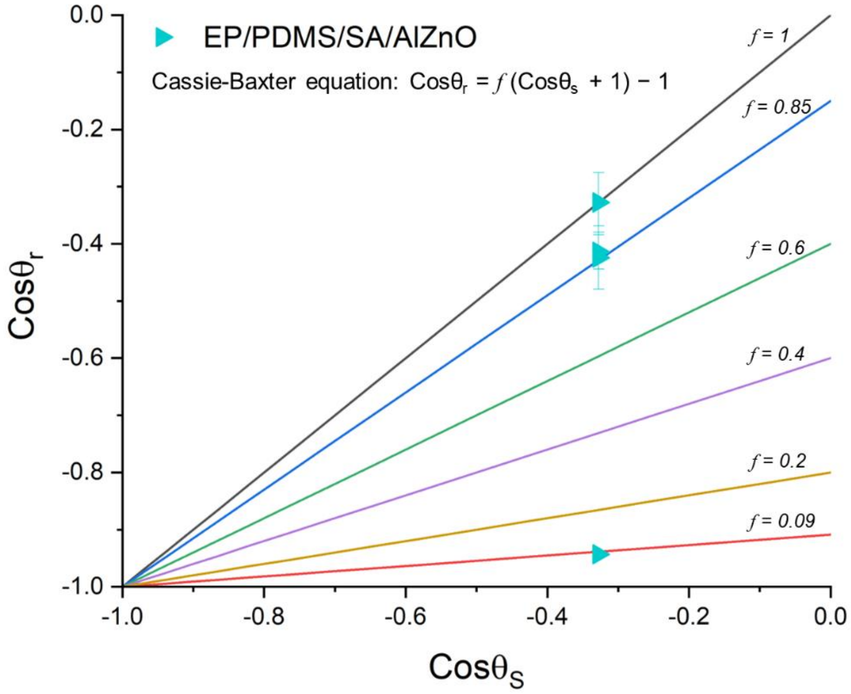

Figure S3. Application of Cassie-Baxter model to changes in the water contact angle of EP/PDMS/SA/AlZnO coating by the increase of surface roughness. In the Cassie-Baxter model, the contact angle for the rough surface ($\mathrm{Cos}\theta_r$) was calculated as a function of liquid/solid contact area (f) for various angles of the smooth surface ($\mathrm{Cos}\theta_s$)
